# International Congress on Tuberculosis

**Published:** 1908-01

**Authors:** 


### INTERNATIONAL CONGRESS ON TUBERCULOSIS.

Active preparations for the International Congress on Tuberculosis to be held in Washington, next September, are under way in other countries. The National Committees for France, Germany, Sweden, Austria, Holland, Greece, Bulgaria, Cuba, Venezuela, Brazil and Costa Rica have organized and have forwarded their membership lists to the Secretary-General. The French Committee has a membership of over three hundred and includes men of prominence in public life as well as in the medical profession. The officers of this committee are president, Dr. Louis Landouzy of the medical faculty of the University of Paris; vice-presidents,—Dr. Faisans of the University of Paris, Prof. Vallee the eminent veterinarian, of Alfort, Dr. F. Bezancon of the University of Paris, and Dr. Le Gendre; secretaries,—Dr. Triboulet, Secretary-General of the last International Congress which was held in Paris three years ago, Dr. Nobecourt, Dr. Leon Bernard, Dr. Dehan, and Dr. Georges Bourgeois; treasurer, M. Masson.

The Secretary of the German Committee Dr. Johannes Nietner, was Secretary-General of the recent International Congress on Hygiene and Demography. Other members of the Committee are Dr. Gotthold Pannwitz, Secretary-General of the International Tuberculosis Association, Dr. B. Frankel, Dr. Ernst von Leyden, Professor emeritus of the University of Berlin, and Dr. Johannes Orth, Professor of Pathology in the University of Berlin.

Dr. N. P. Tenderloo, of Leyden, another well known pathologist is a member of the Committee for Holland. Dr. P.K.Pell, of the University of Amsterdam, is chairman of that committee, and Dr. W.J. von Gorcum of The Hague is the secretary.

Dr. A. Herrera Vegas, the Chairman of the Venezuelan committee is president of the Venezuelan Anti-tuberculosis League and a member of the National Academy of Medicine at Catacas. Dr. P. Acosta Ortiz, the vice-president, is a director of the hospital at Vargas, and Dr. L. Razetti another member of the committee is vice-rector of the University of Venezuela and permanent secretary of the National Academy of Medicine. All of the members of Brazilian committee are actively identified with the anti-tuberculosis movement in that country. The Committee includes Dr. J.J. Azevedo Lima, of Rio Janeiro, President of the Brazilian Anti-tuberculosis League; Dr. Oswaldo Cruz, Director-General of the Department of Public Health; Dr. J.J. Seabra and Dr. Cypriano de Freitas, of Rio de Janeiro.

The president of the Cuban Committee is Dr. Guiteras formerly professor of pathology in the University of Pennsylvania and now at the University of Havana. Dr. J. L. Jacobson the vice-president is president of the Cuban Antituberculosis League.

The secretary is Dr. M. G. Lebredo of Havana. Two well known members of this committee are Dr. Aristides Agramonte, the last surviving member of the famous yellow fever commission of the United States Army, and Dr. Carlos J. Finlay who was recently awarded the Mary McKinsley medal by the Liverpool Association for the Study of Tropical Diseases.

Dr. B. Patrikios, the chairman for the committee for Greece is Secretary of the Department of Health of Greece, and Secretary-General of the Greek Red Cross Society. Dr. Aristote Kouzis, the Secretary is a professor of the University of Athens. Dr. Constant Savas, a member of the Committee is a professor of Hygiene in University of Athens, and is physician to the King of Greece; Dr. P.Manoussos is the principal medical director of the military hospital at Athens; Dr. Kalliontzis is professor of surgery and Dr. Pierre J. Rondopoulo is professor of pathology at the University of Athens.

The Hon. Otto von Printzkold, the Chairman of the Swedish Committee, is the first chamberlain of the Swedish Court. The secretary, Dr. Bertil Buhre, is the president of the great Swedish Anti-tuberculosis League, the largest volunteer association of the kind in existence.

The Costa Rican Committee has named Dr. Louis P. Jiminez chairman, and Dr. Teodoro Picado, of San Jose, secretary. Other members are Dr. Teodoro Prestinary, Dr. Benjamin Hernandez and Dr. Marcos Zunega, all of San Jose.

Three chairmen have been named by the Austrian Committee. They are Prof. Leopold v. Schrotter, of the Medical Faculty of the University of Vienna; Dr. Weichselbaum and Dr. Richard Paltauf, of the Department of pathology of the University of Vienna. The secretaries are Dr. H. v. Schrotter, Dr. L. Teleky and Dr. J. D. Bartel.

Dr. M. Rousseff( director of the Department of Health of Sophia, is president of the Bulgarian committee; Dr. Ivan Oggnainoff, secretary of the Superior Board of Health at Sophia, is Secretary, and the members include Dr. Georghi Zolotovitch, Dr. Ivan Theororoff, director of the Sanatorium for Tuberculosis at Trojan, and Dr. S. A. Valcovitch.

---

### IN GEORGIA.

---

"Why, John, it's as cold here in Georgia as it is in New York."

"Cold! Well, didn't we come south for the winter?"—*December Lippincott's.*

A fecal fistula may be made to heal more quickly by the application of the actual cautery.

Persistent bleeding or irregular prolonged menstruation is very suggestive of uterine fibroids.

Large intraabdominal abscesses are often better drained by making a counter incision in the lumbar region.

Woven silver wire for suture material in a recurrent hernia will often succeed when all other means fail.

Orchitis after an operation for hernia is best relieved by wet or glycerin dressing with elevation of the scrotum.

The blood should be examined in all cases of gangrenous gingivitis for evidences of acute lymphatic leukemia.

Bilaterial swelling of the knee joints without pain, in a child, is due either to syphilis or tuberculosis, more likely the latter.

Swabbing out a sinus filled with exuberant granulations with glycerin will often dehydrate them, making them fresh and healthy.

Stretching the anal sphincter alone will in many instances relieve an intense pruritus or a small prolapse of the anal mucus membrane.

A tumor in the soft parts of the cheek near a tooth cavity is often a dentigerous cyst. If the tumor is hard an odontoma may be diagnosed.

A large tumor supposedly a growth of the ovary may be a retroperitoneal mass, usually a sarcoma, having no connection with the sexual organs.

A moderate prolapse of the rectum with hemorrhoids may possibly be relieved by the treatment of the hemorrhoids with damp and cautery.

*American Journal of Surgery.*

---

Grateful Patient—"Doctor, how can I ever repay you for your kindness to me?"

Doctor—"Doesn't matter, old man. Check, money order or cash."—*British Medical Journal.*

### OULD DOCTOR MAGINN.

The ould doctor had only wan failin'
  It stayed wid him, faith, till he died;
And that was the habit av wearin'
  His darby a thrifle wan side!

And twenty times daily 'twas straightened,
  But try as he would for a year,
Not thinkin' he'd give it a teether
  A thrifle down over wan ear!

It sat him lop-sided and aisy;
  It throubled his kith and his kin—
But ach, 'twas the only thing crooked
  About our ould Doctor Ma'Ginn!

And now that he's gone to his glory—
  Excuse me, a bit av a tear—
Here's twenty to wan that his halo
  Is slantin' down over his ear!

(Arthur Stringer in December Smart Set.)

